# Engagement in Ageless Gym Programs Among Older Adults in Rural Communities: A Retrospective Study on Relationships With Age, Health Conditions, and Proximity to Health Facilities

**DOI:** 10.1155/jare/2608531

**Published:** 2025-06-23

**Authors:** Hung-Pin Chen, Yen-Po Yeh, Dih-Ling Luh

**Affiliations:** ^1^Department of Public Health, Chung Shan Medical University, Taichung City, Taiwan; ^2^Changhua Public Health Bureau, Changhua, Taiwan; ^3^Graduate Institute of Epidemiology and Preventive Medicine, College of Public Health, National Taiwan University, Taipei City, Taiwan; ^4^Department of Family and Community Medicine, Chung Shan Medical University Hospital, Taichung City, Taiwan

**Keywords:** Ageless Gym, community health promotion, elderly exercise

## Abstract

**Background:** To investigate the association between community members' participation in Ageless Gym and their age, chronic disease history, lifestyle, and place of residence, and to analyze the related factors that influence their continuous participation in gym activities and physical fitness improvements.

**Methods:** This study was a retrospective intergenerational study in which 1896 people aged 60 or older, who participated in the integrated community screening in Ershui Township, Changhua County, were analyzed, and the dependent variable was the participation in the Ageless Gym (445 people in total, 23.5%). The independent variables were the most recent screening questionnaire, including chronic disease history, health behaviors, age, and distance from the health center. Cox proportional risk regression modeling has been used as a multivariate variance analysis, and all statistical analyses have been conducted using SAS 9.4.

**Results:** The hypotheses were partially supported: (1) People with a history of diabetes and osteoporosis were 1.62 times more likely to participate in ageless fitness than those without disease, respectively. (2) With regard to lifestyle, those who had regular health checks were 1.54 times more likely to participate in Ageless Gym than those who did not. (3) The probability of participating in ageless gyms is 0.69 and 0.42 times higher for people aged 70 to 79 and 80 than for people aged 60 to 69. (4) Elderly people living far from a health center were 0.67 times more likely to participate in Ageless Gym than those living nearby. (5) Comparison between those who are willing to participate and those who have consistently participated in the Ageless Gym revealed that factors that influence consistent participation include exercise habits, chronic diseases, and emphasis on healthy eating. (6) The fitness of people who regularly participate in the gym has improved considerably.

**Conclusions:** This retrospective study provides insights for promoting exercise among the elderly, emphasizing the relationships between participation, age, health conditions, and proximity to health facilities.

## 1. Introduction

In recent years, the world has faced the serious challenge of aging populations, particularly in rural areas. An aging population places a heavy burden on the medical system and public health [1, 2]. Due to the limited medical resources, transport, and economic constraints of rural areas, it is difficult to obtain adequate medical care [3]. Previous studies have shown that SF-36 scores for older adults in urban areas are significantly higher than in rural area [4]. In rural areas, the proportion of elderly persons is significantly higher and chronic diseases are exacerbated. Consequently, the provision of a professional and appropriate health service for this particular group in order to ensure their physical health has become a priority [5].

Exercise is an important factor in the health of the elderly [6]. Studies have shown that moderate exercise promotes better cardiovascular health, reduces the risk of chronic diseases, and improves mental health [7] and mood in elderly people [8]. The exercise also improves strength, flexibility, balance, and coordination and reduces the risk of falls and fractures [9]. Exercise also slows aging and disability and reduces the risk of death [10, 11].

Rural health care (primary health care) plays a crucial role in public health by providing medical services, preventive care, and health promotion [12]. In response to the aging population, health centers have expanded beyond outpatient care, vaccinations, and screenings to include elderly-focused wellness programs. Many have transformed into health promotion hubs, incorporating exercise and healthcare initiatives to enhance seniors' well-being and reduce the healthcare burden on communities.

Despite the growing interest in health promotion activities in rural areas, there is still a relatively large gap in research into certain factors and their relative impact on the participation of rural elderly people in physical activities. Therefore, the aim of this study is to in-depth study the factors associated with the participation of the elderly in the rural gym, as well as the factors affecting their continued participation and the improvement of the situation after their continued participation.

Ershui Township in Changhua County is a typical agricultural community, where residents focus on agriculture during the day and exercise mainly through community activities or walks around their homes. This study therefore examined the residents' willingness to participate in an elderly gym based on a health center and the associated factors in the city of Ershui. The hypothesis of the study was as follows:

1. Since the Ageless Gym in Ershui Township is based on a health clinic, the objective of the elderly who visit a health clinic is to diagnose chronic diseases, the health clinic encourages the public to participate in the Ageless Gym because “exercise is beneficial for the improvement and stabilization of chronic diseases”, so it is assumed that people with a history of chronic diseases will be more likely to participate in the Ageless Gym.
2. Based on the premise of behavioral coherence, it is assumed that older people with healthier lifestyles (nonsmoking, nondrinking, and healthy diet) are more likely to participate in Ageless Gym.
3. It is assumed that the likelihood of participating in the Ageless Gym decreases with age, as previous studies have shown that elderly people can reduce social participation due to aging and disabilities [13].
4. Although health centers are the most basic health care and public health services in Taiwan, they are not practical for all elderly people in the community, and physical distance is often an important factor in the selection of exercise places for elderly people [14]. This study therefore hypothesized that the closer the distance between the elderly home and health center, the higher the probability of participating in Ageless Gym.
5. This study hypothesizes that individuals with healthier lifestyles are more likely to continue participating in fitness activities, leading to sustained engagement and significant improvements in physical fitness.

## 2. Materials and Methods

### 2.1. Study Design

This is a retrospective intergenerational study to examine factors related to community elders' participation in the Ageless Gym in a health center, factors affecting their continued participation in the Ageless Gym and factors affecting their physical condition after their participation.

### 2.2. Background of the Study

This study was conducted in Ershui Township, Changhua County, and included individuals who participated in community-based integrated screening (2005–2020) and those who visited the health clinic after the opening of the Ageless Gym (2017–2021).

Since 2005, the Changhua County Health Bureau has been offering integrated community screening services for many chronic diseases (such as hypertension, diabetes, kidney disease, high blood pressure, and hepatitis B and C) and four major cancers (brain, colorectal, cervical, and oral cancer). During the screening process, participants are also asked about their health-related lifestyles, which serves as a basis for continuing health promotion services. This is very important for the public health of Changhua County. The screening program is held in 26 towns in Changhua County, and about 400 people in Ershui Township participate annually.

Ershui Township has the highest proportion of elderly in Changhua County, with 26.7% of the population aged 65 or older [15]. The Ershui Township Health Clinic is a rural health organization located in the city area of Ershui Township that provides outpatient medical services in addition to public health services. The monthly ambulatory capacity of the clinic is approximately 1,600, of which about 58% are over 65 years old, and the annual number of elderly people attending the clinic covers approximately 40% of the over 65-year-old population of the Township of Ershui.

In order to further promote healthy lifestyles, Ershui Township Health Clinic opened Ageless Gym in 2017. The Ageless Gym, opened in 2017, provides supervised exercise programs tailored for elderly participants, incorporating resistance and cardiovascular training.

The Health Center actively promotes the elderly and chronically ill to participate in functional fitness testing and gym training. This is an attempt to help the elderly and chronically ill to understand and improve their physical fitness status, thereby improving their health conditions. The fitness test items of the Ageless Gym include body composition (height, weight, triceps sebum thickness, waist circumference, hip circumference), flexibility (back grip with both hands, forward bending in a chair), muscular strength and endurance (handgrip strength, upper limbs: 30 s for flexion bells, lower limbs: 30 s for sitting and standing), cardiorespiratory endurance (2-min stepping), and dynamic balance and agility (2.44-m standing up and walking around), static balance (standing on one foot).

### 2.3. Subjects of the Study and Sources of Information

**Figure 1:**
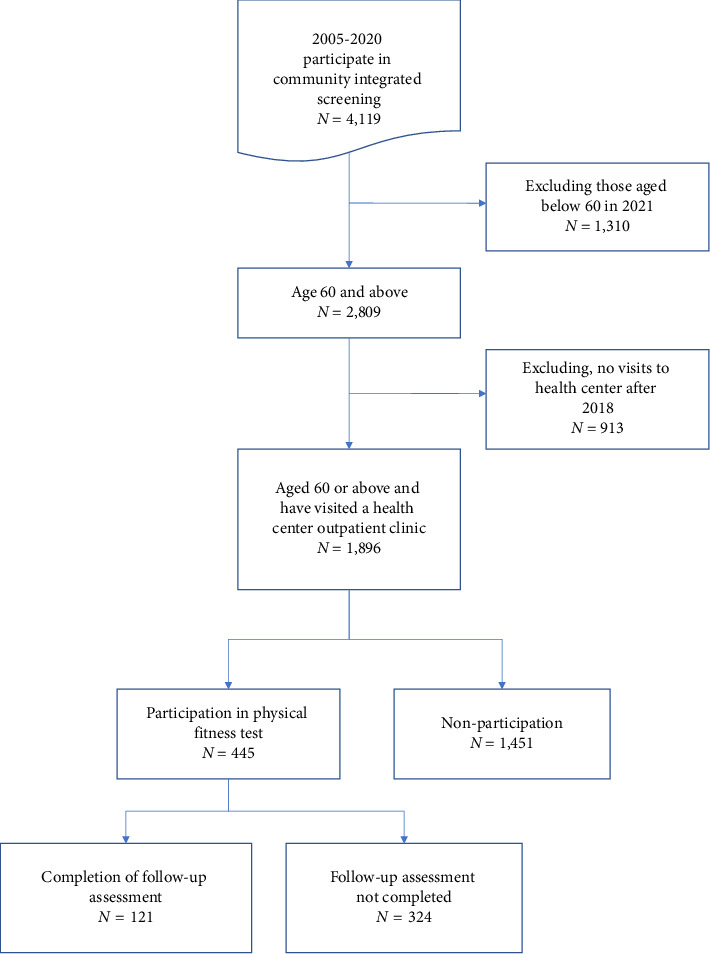
Flowchart for screening and grouping of study participants.

The data for this study were obtained from Changhua County Community Integrated Screening Database, Ershui Township Health Clinic Medical Outpatient Information, and Ageless Gym records. The study included 7046 records (4119 individuals) who participated in the Ershui Township screening of the Changhua County Community Integrated Selection Database between 2005 and 2020. Since the region is rural and vulnerable to aging, persons over the age of 60 between 2017 and 2021 (*n* = 2809) were included in the study, and the socio-demographic and lifestyle data from the screening questionnaire will be used. Variable description: For the gym database, the Ageless Gym attendance data from 2017 to 2021 (*n* = 803) were used. Since Ageless Gym is based on the platform of health clinics and outpatient clinics, and health clinic doctors suggest that patients receive fitness tests and participate in Ageless Gym during outpatient clinic visits, the criteria for inclusion of this study include: (1) participating in the Changhua Integrated Screening Service; (2) being a patient of the Ershui Township Health Clinic; and (3) being a patient of the Ershui Township Health Clinic; (4) being a patient of the Changhua Health Clinic; and (5) being a patient of the Changhua Health Clinic. The criteria for exclusion were: those who had not been seen by the health center since its opening (because they had not been recommended by the doctors of the health canter); a total of 1896 subjects were finally enrolled in the study. Of these subjects, 445 had participated in the “Ageless Gym” programmed and 1451 did not participate. A total of 324 (72.8%) did not participate in the examination and 121 (27.2%) continued to participate in the examination and the second examination (Figure 1).

### 2.4. Definition of Variables

For the purpose of this study, part I: “willingness to participate in the Ageless Gym” is used as a variable based on the Ageless Gym database, and those who have fitness test records are considered to be “will”.

To explore factors associated with participation in the Ageless Gym, data from the Changhua Integrated Screening Database were collected, including socio-demographic variables, lifestyles, disease history, and current results of chronic diseases, as follows: socio-demographic variables include gender (male, female), age (categorized as 60–69, 70–79, and 80+), education level (under primary school, middle & high school, and above university). Healthy lifestyle variables include smoking (none, quit, currently smoke), alcohol use (never, quit, currently drink, occasionally), and physical activity (none, less than 150 min per week, 150 min or more per week). According to the National Health Service (NHS) Healthy Eating Guidelines, dietary behavior is classified into two categories and is considered healthy if it meets the criteria. Dietary habits included vegetables (more than two bowls per day were considered healthy) and dairy products (including dairy products, soybeans or yogurt, seven times per week). Participants self-reported their chronic disease histories, including: high blood pressure (no, yes), diabetes (no, yes), heart disease (no, yes), liver disease (no, yes), kidney disease (no, yes), stroke (no, yes), asthma (no, yes), and osteoporosis (no, yes). Health screening frequency and history were recorded based on self-reported data and integrated screening records. The results of the current health examination include blood pressure (considered abnormal when systolic blood pressure is ≥ 130 mmHg or diastolic blood pressure is ≥ 85 mmHg or if high blood pressure medication has been used), blood glucose (considered abnormal when fast blood glucose is ≥ 100 mg/dL or if diabetic drugs have been used), cholesterol (considered abnormal when cholesterol is ≥ 150 mg/dL or if high cholesterol drugs have been used), waist circumference (body obesity whose circumference is ≥ 90 cm for men and ≥ 80 cm for women as abnormal), and HDL (HDL < 40 mg/dL for men and < 50 mg/dL for women as abnormal).

The data in the screening database were divided and the screening data closest to the date of joining Ageless Gym were used in this study. For those who have not joined the Ageless Gym, the latest screening data have been used as analyzed data.

Proximity to the health center was categorized into three groups (within 1000 m, 1000–3000 m, and beyond 3000 m) using Google Maps estimates.

The second part is divided into two groups: “willingness” and “continuous participation”. The physical fitness test included seven items, such as “Hand Grip Strength”, “Flexed Abdominal Bells”, “Lower Limb Sitting and Standing”, “Back Grasping”, “Forward Bending”, “Two-Minute Stepping”, and “Getting Up and Walking Around”, and were assessed as “Poor”, “Fair”, “Average”, “Good”, and “Excellent”, and quantified in ascending order as scales of 2–10. The results of “BMI”, “triceps fat thickness”, and “standing on a leg” were classified as “Poor”, “Average”, and “Excellent” and quantified in the order of 2, 6, and 10.

The third part was the improvement in physical fitness through participation in the Ageless Gym, and the dependent variables were the test scores of participation in the Ageless Gym, and the independent variables included the test interval (< 12 months vs. ≥ 12 months), gender (male vs. female), age (≥ 70 vs. 60–69), level of education (middle school and above vs. below elementary school), exercise (< 150 min/week vs. none, ≥ 150 min/week vs. none), diet (mount of vegetables: 2 bowls/day vs. less than 2 bowls/day; dairy products: 7 portions/week vs. less than 7 portions/week), and health status (diabetes mellitus: yes vs. none; peripheral vascular diseases: yes vs. none; and number of chronic diseases: 1 vs. none, 2+ vs. none). There are no compulsory rules for participants in Ageless Gym. A second test is carried out when the participant has been trained for more than 3 months. As a result, the interval between the first and second tests was relatively short for participants who had undergone regular training, while the interval between the first and second tests was relatively long for participants who had not undergone regular training. In this study, the time between the first and second tests was divided into two groups: less than 12 months and more than 12 months to distinguish the regularity of training and the effect on physical fitness of participants. These variable definitions are useful to analyze factors associated with participation in ageless gyms and to further understand the physical fitness status of older adults who participate and continue to participate in ageless gyms and the factors influencing them.

### 2.5. Statistical Analysis

The descriptive statistics of this study included frequency and percentage. Since the time between screening and participation in the Ageless Gym varied from person to person, Cox regression models were used to present the relationship between each variable and participation in the Ageless Gym, and the time was calculated according to the following principle: for those who participated in the Ageless Gym, the time was calculated from the date of screening to the date of participation in the gym, which was closest to the date of screening. For those who participated in the Ageless Gym, the calculation was based on the date of screening closest to the date of joining the gym. For those who do not participate in the gym, the time calculated is until December 31, 2021, based on the screening date closest to the opening time of the gym. Variables for the Cox proportional hazards model were selected based on theoretical relevance and statistical significance (*p* < 0.05) in univariate analysis to balance explanatory power and model simplicity. Key socio-demographic factors such as age and education were included based on their established importance in prior research. Additionally, chronic disease histories (e.g., diabetes, osteoporosis) and lifestyle factors (e.g., exercise frequency, vegetable consumption) were incorporated due to their potential influence on participation. Screening results (e.g., BMI, blood pressure, glucose levels, waist circumference, triglycerides, HDL), distance to gyms, and integrated screening frequency were included as relevant health indicators. Variables excluded from the multivariate model (e.g., hypertension, heart disease) were those that did not meet the statistical significance threshold in univariate analyses or lacked sufficient theoretical support for a direct relationship with participation.

Univariate analyses (Table 1) were conducted as a preliminary screening step to identify candidate variables for multivariable analysis. These analyses included chi-square tests and independent sample *t*-tests to examine associations between individual variables and gym participation. As these analyses are exploratory and guide the selection of variables for subsequent multivariable modeling (Table 2), no formal corrections for multiple comparisons (e.g., Bonferroni adjustment) were applied. Final inferences were drawn from the multivariable Cox regression model and mixed-effects model (MEM), which simultaneously accounted for multiple variables and confounding factors. To examine potential interaction effects, interaction terms (age × diabetes, age × osteoporosis, age × hypertension, and age × chronic diseases) were included in the multivariate Cox regression model (Table 2) and MEM (Table 3). These interaction terms were tested to assess whether the effects of chronic disease status on gym participation and fitness outcomes varied across age groups.

The assumptions of the Cox proportional hazards model were tested to ensure the validity of the analysis. The proportional hazards assumption was evaluated using Schoenfeld residuals, and no significant violations were observed. In addition, the linearity assumption for continuous covariates was assessed by testing for nonlinearity, and all included variables met the required assumptions.

An MEM was used to assess the impact of gym participation on physical fitness, with fitness score as the dependent variable. Random intercepts accounted for individual variability in baseline fitness. Fixed effects included demographic (gender, age, education), lifestyle (exercise, diet), and health factors (chronic diseases) due to their established influence on fitness outcomes in elderly populations. In addition to comparing initial and follow-up fitness scores, baseline fitness levels were included as covariates in the MEM to control for preexisting differences among participants. Other potential confounders, such as age, gender, exercise habits, and chronic disease history, were also adjusted for in the analysis. This approach ensures that the observed fitness improvements are more robustly attributed to gym participation rather than underlying differences in baseline characteristics.

All statistical analyses were performed using two-tailed tests and the significance level was set at *p* < 0.05. Statistical analyses were performed using SAS 9.4 statistical software.

### 2.6. Ethical Aspects

During the Chung Shan Medical University Hospital Institutional Review Board review process, the data collected for this study were determined to be part of program implementation and evaluation, and specific informed consent was not required by clients (CSMUH-CS1-22173). All data were deidentified to protect patient confidentiality.

## 3. Results

### 3.1. Description of the Basic Information of the Research Subjects

A total of 1896 Ershui Township residents who participated in the integrated screening were enrolled in this study. In terms of gender distribution, 57.8% were women, 79.9% were between 60 and 69 years old, and 55.4% were under primary school age. In terms of lifestyle, 88.9% of the participants did not smoke, 89.4% did not drink alcohol, 50.24% did not exercise, 85.1% did not have diabetes, and 79.2% did not have hypertension. More than 2 chronic diseases and 1 chronic disease accounted for 35.2% and 16.1%, respectively. Vegetable intake was 89.8% healthy, dairy intake was 74.9% unhealthy, egg intake was 92.7% unhealthy, and fruit intake was 67.5% unhealthy. With regard to screening results, 56.6% had a BMI of 24 or greater. The percentage of participants who had not undergone health checks within a year was 92.7% (Table 4).

### 3.2. Factors Related to the Willingness of the Elderly in the Community to Participate in the Ageless Gym

The univariate analyses (Table 1) identified significant associations between gym participation and factors such as age, gender, education, exercise habits, and chronic disease history. These results were exploratory and were used to guide the selection of variables for the multivariable Cox regression model (Table 2). Final conclusions were based on multivariable analyses, which mitigate the issue of multiple comparisons inherent in univariate testing.

The interaction terms between age and chronic disease history (age × diabetes, age × osteoporosis, age × hypertension, and age × chronic diseases) were tested in the Cox regression model and MEM. No significant interaction effects were observed, indicating that age and chronic disease status independently influenced participation in the Ageless Gym and fitness outcomes (Tables 2 and 3). A Cox multivariate regression analysis (Table 2) was conducted using sociodemographic variables (including gender, age, and educational level) and the above variables that were important in Cox's single-variable analysis as independent variables, and participation status in the elderly fitness room as dependent variables, and it was found that: the probability of joining a fitness room for the elderly for those aged 70–79 and 80 or older was 0.69 and 0.42 times that of those aged 60–69, and the probability of joining a fitness room for the elderly people with university education were 1.52 times more likely to participate in ageless gyms than people with primary education or less. People with more than 150 min of exercise a week are 1.31 times more likely to participate in Ageless Gym than those without exercise, people with diabetes are 1.62 times more likely to participate in an Ageless Gym than those without diabetes, and people with osteoporosis are 1.62 times more likely to participate in Ageless Gym than those without osteoporosis. Those with abdominal obesity were 1.33 times more likely to attend the antiaging fitness program than those without abdominal obesity, and those living in a remote village were 0.67 times more likely to attend the antiaging fitness program than those living in an adjacent village. Those who had a physical examination in 1 year were 1.54 times more likely to participate in the ageless gym than those who had not had a physical examination in 1 year, and those who had participated in 2, 3, or more integrated health examinations were 1.95, 3.02, or 6.41 times more likely to participate in the ageless gym than those who had participated in one integrated health examination.

### 3.3. Relevant Factors Affecting the Sustainability of the Ageless Gym for the Elderly in the Community

This study compared “willing” (first tests) and “continuing” participants (second tests), and found that young age, exercise habits, chronic diseases, diabetes, and the emphasis on healthy diet (vegetables, dairy products) were factors affecting the ability to continue participating in the gym (Table 5).

The proportion of those who exercised 150 min a week was significantly higher among consistent participants than among willing participants (*p* = 0.0018). Diabetics, people with chronic diseases, and a healthy diet (food, dairy products) were significantly more likely to be “consistent participants” than “willing participants”.

### 3.4. Effectiveness of Continuous Participation in the Ageless Gym

To evaluate the effectiveness of participating in the ageless gym, the present study analyzed the factors that influence the fitness scores using a MEM, which is presented in Table 3. First, the duration of the testing period had a significant effect on the fitness scores, and the fitness scores of subjects with a 12-month interval were significantly higher than those of subjects with a 12-month interval or more (estimated = 3.43, *p* = 0.0099).

MEM analysis showed that women, younger age groups, and those with higher education had significantly higher fitness scores (*p* < 0.05). In addition, exercise and dietary habits have also had a significant impact on fitness scores. Participants who exercised for more than 150 min a week had significantly higher fitness scores than those who did not (estimate = 7.29, *p* = 0.0114), and those who consumed more than two vegetables a day had significantly higher fitness scores than those who ate fewer (estimate = 22.38, *p* = 0.0038).

With respect to health, subjects with a history of diabetes had a significantly lower fitness score than those without diabetes (estimate = −10.54, *p* = 0.0002) and subjects with a chronic disease had a significantly higher fitness score than those without chronic disease (estimate = 11.02, *p* = 0.0026). These results indicate that regular fitness activities are effective in improving the physical condition of the elderly, especially for those with a healthier lifestyle (e.g. regular exercise and healthy eating habits). On the other hand, diabetics have lower fitness and may need additional health support and exercise guidance.

## 4. Discussion

The following is a discussion of each research hypothesis.

### 4.1. Hypothesis 1. Those With Chronic Diseases Are More Likely to Participate in the Ageless Gym

Since the Ageless Gym in Ershui Township is based on a health clinic and most visits to the health clinic are for chronic diseases, the health clinic often uses the reason that “exercise is beneficial to improving and stabilizing chronic diseases” as a reason to encourage the public to participate in the Ageless Gym, and therefore, it is assumed that those who have a history of chronic diseases are more likely to participate in the Ageless Gym. The results of the study “support in part” the hypothesis that people with self-declared diabetes mellitus and osteoporosis are more likely to participate in Ageless Gym. However, there is no statistically significant relationship between self-reported hypertension, heart disease, liver disease, kidney disease, stroke, asthma, and participation in Ageless Gym.

Previous studies have shown mixed results regarding chronic disease history and exercise behavior, with some suggesting increased activity after diagnosis and others finding no significant change [16]. In short, the results of this study echo the results of previous qualitative studies on the history of chronic disease history and exercise behavior among the elderly: the elderly cannot participate in exercise programs because of the physical limitations of their disease, but they can also be motivated to exercise to improve their physical and mental health [17].

This study found that older diabetics and individuals with osteoporosis were more likely to participate in the gym. While this is partially attributed to health education provided at the health center, other potential factors should also be considered. For diabetic patients, the need to manage blood glucose levels may create a stronger motivation to engage in exercise programs [18]. Additionally, access to regular consultations with healthcare providers at the center may facilitate gym participation through personalized encouragement and monitoring. For osteoporosis patients, participation may be driven by a desire to improve bone density and reduce the risk of fractures, as exercise—particularly resistance training—is widely recommended for these patients [19]. Psychological factors, such as self-efficacy and perceived benefits of exercise, may also play a role but were not evaluated in this study. Further research could investigate these factors, as well as the role of disease severity and comorbidities, in influencing participation rates among patients with chronic diseases.

### 4.2. Hypothesis 2. Seniors With Healthier Lifestyles Are More Likely to Participate in the Ageless Gym

The results of this study “in part support” the hypothesis that only regular exercise, nonsmokers and repeat community screening volunteers in the multivariate model are more likely to participate in Ageless Gyms, which is consistent with this hypothesis and previous research [20]. However, alcohol consumption, almond consumption, and all food-related behaviors were not significantly associated with participation in the Ageless Gym, which not only did not support the hypothesis of this study but also contradicted the results of previous studies: the analysis of BRFSS data in the United States showed that people who consumed more alcohol spent more time exercising [21], and adults with a healthier diet have higher levels of physical activity [22]. Elderly people were more likely to enter a gym the more times they were screened: the number of screenings and the number of health checks during the screening year were positively related to the probability of entering Ageless Gym. The likelihood of attending a gym increased significantly as the number of tests increased. Elderly people who participate in regular health screenings may take their health more seriously and are therefore more likely to participate in fitness activities [23].

Regular exercise and nonsmoking were positively associated with gym participation, but no significant relationship was found with alcohol consumption or diet, possibly due to differences in lifestyle correlations among rural elderly populations. Further research is needed to explore these inconsistencies [24].

### 4.3. Hypothesis 3. The Likelihood of Participating in an Ageless Gym Decreases With Age

The results of this study support the hypothesis that the probability of participation in Ageless Gym decreases with age. According to the results of previous research, older adults are less likely to exercise as they get older [25]. The observed decline in participation among older age groups is likely influenced by multiple factors. Physical limitations, such as decreased mobility, chronic pain, or frailty, may discourage older adults from participating in gym-based activities [26]. Social factors such as lack of social support, including feeling uncomfortable going out alone and no meaningful social connections, may stop older adults from joining sports or community exercise programs. Additionally, lack of knowledge about the benefits of exercise, fear, and unfamiliarity with the gym environment may further hinder gym participation [27]. Environmental and logistical challenges, such as transportation difficulties and accessibility issues within the gym, could disproportionately affect older individuals. These findings align with previous research [28] suggesting that age-related declines in participation are multifactorial, involving physical, social, psychological, and environmental factors.

### 4.4. Hypothesis 4. Correlation Between Residence and Gym Distance

The results of this study support the hypothesis that seniors who live near Ageless Gym are much more likely to participate. This conclusion is consistent with previous research, *which shows that closer proximity to fitness facilities increases participation rates* [29]. The findings of this study underscore that geographic distance serves as a significant barrier to gym participation among older adults. Addressing this issue requires the implementation of targeted strategies aimed at increasing access for individuals in remote areas. For example, establishing compact fitness facilities within community elder care centers and deploying exercise equipment alongside professional trainers to underserved villages can effectively mitigate the impact of distance [30]. Additionally, promoting government-subsidized transportation programs, such as senior-friendly taxi services, can facilitate easier access to fitness facilities like the Ageless Gym [31]. Furthermore, leveraging digital technology to develop at-home exercise solutions—including instructional videos, simple exercise tools, and personalized home workout plans—can help overcome participation barriers linked to geographic isolation [32].

### 4.5. Hypothesis 5. People Who Lead Healthier Lifestyles Are More Likely to Continue Exercising in the Gym

The results of this study show that people who have a healthier diet and a lifestyle of exercise can maintain their participation in gym exercise. The study also reported that healthy habits such as regular exercise and balanced diet can help maintain exercise [33].

### 4.6. Hypothesis 6. Continued Participation in the Ageless Gym Will Lead to Improved Physical Fitness

Recent research has shown that regular participation in resistance training programs has significant benefits not only for improving muscle strength but also for improving general functional abilities [34]. In the case of the Ageless Gym, which is not compulsory for the elderly, the elderly use their free time to train in the health canter's Gym, as well as their agricultural or household tasks. Even in this case, elderly people are able to progress in the second test. The results show that joining a gym is effective in improving physical condition.

The study's findings should be interpreted with caution due to the selection bias introduced by the inclusion criteria. The reliance on health screening participants may overestimate participation rates and the health benefits of the Ageless Gym, as these individuals are likely to have higher baseline health awareness. Efforts to expand access to fitness programs for less health-conscious or underserved populations are necessary to address these disparities.

This long-term study does not account for temporal influences like external events or operational changes. While national health policies consistently promoted elderly exercise, gym closures during COVID-19 likely affected participation trends. The lack of research on Taiwan's societal attitudes toward exercise is a limitation. Future studies should include qualitative assessments or national surveys to address this gap. Segmenting analysis by time periods could clarify participation fluctuations and external influences. Longitudinal interviews may also help understand motivations and barriers to gym engagement over time.

The use of self-reported data introduces limitations that should be considered when interpreting the results. Recall and social desirability biases may have led to inaccuracies in reported lifestyle behaviors, potentially impacting the observed associations with participation in the Ageless Gym. Future research could incorporate objective measures, such as biochemical markers for smoking or alcohol use and food frequency questionnaires validated against dietary biomarkers, to enhance the reliability of the data.

The study accounted for baseline fitness levels and key confounders, such as exercise habits and chronic disease status, to strengthen the validity of the findings. By including these variables in the MEM, the analysis minimizes bias and provides a clearer understanding of how gym participation contributes to fitness improvements. However, some unmeasured factors, such as adherence to exercise regimens and training intensity, were not captured, which could influence the outcomes.

The MEM incorporated random intercepts to account for unobserved heterogeneity among individuals, ensuring that differences in baseline fitness levels were adequately controlled. The inclusion of fixed effects, such as demographic characteristics, lifestyle factors, and chronic disease history, was guided by both theoretical relevance and prior evidence linking these factors to physical fitness outcomes. This approach provides a more nuanced understanding of how these covariates contribute to fitness improvements over time.

This study employed univariate analyses as an initial step to screen variables associated with gym participation. While these exploratory analyses involved multiple comparisons, corrections were not applied because they were not used for final inference. Instead, the multivariable Cox regression model (Table 2) and MEM (Table 3) provided adjusted estimates accounting for multiple variables and confounding factors. Future studies could consider formal corrections for multiple comparisons during univariate screening to further enhance robustness [35].

### 4.7. Research Limitations and Recommendations

The main limitations and recommendations of the study are summarized as follows.

1. This study is limited by selection bias inherent in its design, as the population is restricted to individuals who participated in community health screenings and visited the health clinic. Older adults who are less health-conscious or unable to access these services may be underrepresented, which could limit the generalizability of the findings. These excluded populations may exhibit different characteristics, such as lower levels of physical activity or greater barriers to participation. Future research should explore methods to include these groups, such as outreach to homebound individuals or those in underserved areas.
2. Limitations on the use of secondary data: The study used questionnaire records of Changhua County integrated screening to conduct the analysis, which was not intended to focus on the willingness to participate in the gym, and could not cover in a comprehensive way the possible influencing factors of participation in the gym.
3. This study is limited by selection bias inherent in its design, as the population is restricted to individuals who participated in community health screenings and visited the health clinic. Older adults who are less health-conscious or unable to access these services may be underrepresented [36], which could limit the generalizability of the findings. These excluded populations may exhibit different characteristics, such as lower levels of physical activity or greater barriers to participation [37]. Future research should explore methods to include these groups, such as outreach to homebound individuals or those in underserved areas.
4. The measurement of proximity in this study is based on distances calculated using Google Maps, which reflects the actual travel distance along roads rather than straight-line distances. While this method provides a more practical approximation, it does not fully account for real-world barriers, such as varying road conditions, traffic, or the availability of transportation options. These factors could significantly impact participation in the Ageless Gym, particularly for individuals without personal vehicles or access to reliable public transportation. Future studies should incorporate additional measures, such as estimated travel time or accessibility indices to better capture the practical challenges of reaching health facilities and their influence on gym participation.

This study relies on self-reported data for lifestyle variables, including smoking, alcohol use, and dietary habits. Self-reported data are subject to recall bias, where participants may inaccurately remember or report past behaviors, and social desirability bias, where responses may be influenced by the desire to present oneself in a favorable light. These biases could affect the accuracy of the findings, particularly for lifestyle-related variables. For example, participants may underreport unhealthy behaviors, such as smoking or alcohol consumption, or overreport adherence to healthy dietary habits.

The observed improvements in fitness scores among participants who consistently attended the Ageless Gym have meaningful clinical implications. Increased muscle strength, balance, and flexibility, as indicated by better performance in fitness tests, are associated with a reduced risk of falls and improved functional independence in older adults. For instance, enhanced lower limb strength and dynamic balance can directly translate to better mobility, reducing the likelihood of fall-related injuries, which are a leading cause of morbidity in this population [38]. Furthermore, these fitness improvements may contribute to broader health benefits, such as enhanced cardiovascular health, better management of chronic conditions (e.g., diabetes, hypertension), and improved quality of life. Regular participation in structured exercise programs like the Ageless Gym can play a vital role in delaying functional decline and supporting healthy aging [32].

This study is based on data collected from a single rural township (Ershui Township) in Taiwan, which limits the generalizability of the findings to other settings. The socio-demographic, cultural, and environmental characteristics of Ershui Township may differ from those in urban areas or other rural regions, potentially influencing participation in gym activities and associated fitness improvements. For example, differences in access to healthcare facilities, transportation infrastructure, or community engagement might yield different outcomes in other populations. While this study provides valuable insights into the factors influencing gym participation among older adults in rural areas, caution should be exercised when applying these findings to other contexts. Future research should include diverse geographic and socioeconomic settings to better understand the broader applicability of these results.

This study relies on secondary data collected during health screenings, which introduces inherent limitations. The data were not originally designed to examine gym participation, and therefore, may not capture all relevant factors influencing participation, such as psychological motivators, social support, or attitudes toward exercise. Additionally, data on adherence to gym activities and exercise intensity were not available, which could further refine the understanding of participation behavior and outcomes.

Another limitation is the reliance on self-reported data for certain variables, which may introduce recall and social desirability biases. Despite these limitations, the study provides valuable insights into factors influencing gym participation in a rural context. Future studies should consider incorporating primary data collection to capture a broader range of variables, including qualitative aspects such as motivation and barriers.

### 4.8. Research Implication and Contribution

1. To encourage the public to participate in the Ageless Gym, 23.5% of the elderly who participate in the community integrated health screening choose to participate in the Ageless Gym program. After the first examination, 27.2% of elderly people continued to exercise without being forced to exercise at Ageless Gym.
2. The main factors influencing the participation of the elderly in the ageless gym are age, the presence of diabetes or osteoporosis, the distance from the gym, and regular health examinations.
3. This retrospective study demonstrates that participation in the Ageless Gym is associated with improvements in physical fitness among older adults. The findings highlight the importance of promoting regular exercise through accessible fitness programs to delay physical aging and improve public health outcomes.
4. The delay in physical aging and functional decline of the elderly is one of the most important problems in public health. The study provides information on factors affecting the participation of the elderly in fitness centers, facilitates the design of targeted programmers for the participation of the elderly in the future, and provides important guidelines to policy makers.
5. The findings highlight the importance of promoting structured exercise programs for older adults, particularly in rural areas. The observed fitness improvements not only address physical health but may also have downstream effects on psychological well-being [32], social participation, and healthcare cost reduction by preventing injuries and disabilities [39]. Policymakers and healthcare providers should integrate such programs into community health promotion strategies.

## Figures and Tables

**Table 1:** Factors associated with participation in Ageless Gym: results of univariate Cox regression analysis.

Variable	Hazard ratio	95% confidence interval
Demographic variables		
Gender		
Female	1	
Male	0.92	0.76–1.12
Age		
60–69	1	
70–79	0.75	0.62–1.02
80+	0.47	0.24–0.92
Education level		
Elementary school and below	1	
Middle and high school	1.35	1.09–1.67
College and above	1.92	1.39–2.65
Lifestyle habits		
Smoking		
Never	1	
Quit	0.97	0.72–1.30
Current smoker	0.49	0.34–0.72
Alcohol consumption		
Never	1	
Alcohol consumption	0.81	0.45–1.44
Current drinker	0.94	0.67–1.33
Occasional drinker	0.96	0.71–1.30
Betel nut chewing		
Never	1	
Quit	0.81	0.56–1.16
Current chewer	0.67	0.32–1.42
Exercise		
None	1	
< 150 min/week	1.25	0.99–1.58
≥ 150 min/week	1.15	0.92–1.44
Health status		
Diabetes		
Yes versus No	1.85	1.48–2.32
Hypertension		
Yes versus No	1.43	1.18–1.73
Heart disease		
Yes versus No	1.12	0.82–1.53
Hepatitis		
Yes versus No	1.23	0.73–2.06
Kidney disease		
Yes versus No	1.60	0.88–2.91
Stroke		
Yes versus No	0.54	0.22–1.29
Osteoporosis		
Yes versus No	2.08	1.51–2.87
Asthma		
Yes versus No	0.85	0.38–1.91
Hyperlipidemia		
Yes versus No	1.40	0.99–1.97
Chronic diseases		
None	1	
1 condition	1.32	1.01–1.73
≥ 2 conditions	1.50	1.22–1.85
Dietary habits		
Vegetable intake		
≥ 2 bowls/day versus < 2 bowls/day	1.86	1.29–2.69
Dairy products		
≥ 7 times/week versus < 7 times/week	0.72	0.58–0.91
Egg products		
≥ 7 times/week versus < 7 times/week	1.06	0.72–1.57
Fruits		
≥ 7 times/week versus < 7 times/week	1.06	0.86–1.29
Screening tests		
BMI		
< 24	1	
≥ 24	1.26	1.02–1.55
Blood pressure		
Normal	1	
Abnormal	1.63	1.26–2.11
Blood glucose		
Normal	1	
Abnormal	1.52	1.26–1.84
Waist circumference		
Normal	1	
Abnormal	1.46	1.19–1.79
Triglycerides		
Normal	1	
Abnormal	1.23	1.01–1.50
High-density lipoprotein		
Normal	1	
Abnormal	1.30	1.08–1.58
Metabolic syndrome		
No	1	
Yes	1.47	1.21–1.78
Others		
Distance from health center		
Adjacent village	1	
Nearby village	0.78	0.62–0.98
Distant village	0.56	0.44–0.71
Health checkup within 1 year		
No	1	
Yes	1.72	1.26–2.35
Participation in comprehensive screening		
Once	1	
Twice	2.09	1.64–2.68
Thrice	3.50	2.61–4.70
Four or more	6.81	5.05–9.18

**Table 2:** Factors associated with participation in the elderly fitness program: results of Cox regression multivariate analysis.

Variable	Hazard ratio	95% confidence interval
Demographic variables		
Gender		
Female	1	
Male	1.08	0.82–1.41
Age		
60–69	1	
70–79	0.69	0.53–0.91
≥ 80	0.42	0.21–0.83
Education level		
Elementary school and above	1	
Middle and high school	1.06	0.84–1.35
College and above	1.52	1.06–2.19
Lifestyle habits		
Smoking		
Never	1	
Quit	0.96	0.66–1.39
Current smoker	0.58	0.37–0.91
Alcohol consumption		
Never	1	
Alcohol consumption	0.93	0.49–1.74
Current drinker	0.94	0.62–1.41
Occasional drinker	1.31	0.94–1.83
Exercise		
None	1	
< 150 min/week	1.19	0.94–1.51
≥ 150 min/week	1.31	1.02–1.67
Health status		
Diabetes		
Yes versus No	1.62	1.20–2.19
Hypertension		
Yes versus No	1.20	0.86–1.68
Osteoporosis		
Yes versus No	1.62	1.14–2.31
Chronic diseases		
None	1	
1 condition	0.95	0.69–1.29
2 or more conditions	0.91	0.61–1.35
Dietary habits		
Vegetable intake		
≥ 2 bowls/day versus < 2 bowls/day	1.17	0.80–1.72
Dairy products		
≥ 7 times/week versus < 7 times/week	1.03	0.81–1.30
Screening tests		
BMI		
< 24	1	
≥ 24	0.96	0.74–1.23
Blood pressure		
Normal	1	
Abnormal	1.27	0.95–1.70
Blood glucose		
Normal	1	
Abnormal	1.14	0.91–1.44
Waist circumference		
Normal	1	
Abnormal	1.33	1.02–1.73
Triglycerides		
Normal	1	
Abnormal	0.96	0.77–1.20
High-density lipoprotein		
Normal	1	
Abnormal	0.99	0.80–1.23
Others		
Distance from health center		
Adjacent village	1	
Nearby village	0.98	0.77–1.24
Distant village	0.67	0.52–0.86
Health checkup within 1 year		
No	1	
Yes	1.54	1.12–2.12
Participation in comprehensive screening		
Once	1	
Twice	1.95	1.50–2.54
Thrice	3.02	2.20–4.14
Four or more times	6.41	4.60–8.93

*Note:* Interaction terms (age × diabetes, age × osteoporosis, age × hypertension, and age × chronic diseases) were tested in the multivariate Cox model. No significant interactions were found; therefore, they were not retained in the final model.

**Table 3:** Factors affecting fitness scores.

Variable	Estimate	*p* value
Assessment interval		
< 12 months versus ≥ 12 months	3.43	0.0099
Demographics		
Gender		
Male versus female	−5.29	0.042
Age		
≥ 70 versus 60–69	7.18	0.0115
Education		
≥ Middle versus ≤ elementary	4.95	0.0417
Exercise		
< 150 min/week versus none	4.73	0.1312
≥ 150 min/week versus none	7.29	0.0114
Diet		
Vegetable intake		
≥ 2 bowls/day versus < 2 bowls/day	22.38	0.0006
Dairy intake		
≥ 7 times/week versus < 7 times/week	1.00	0.7038
Health status		
Diabetes		
Yes versus no	−10.54	0.0002
Osteoporosis		
Yes versus no	−3.06	0.4652
Chronic diseases		
1 versus none	11.02	0.0026
≥ 2 versus none	1.91	0.5457

*Note:* Interaction terms between age and chronic disease status (age × diabetes, age × osteoporosis, and age × chronic diseases) were tested but did not show significant effects. Therefore, they were not retained in the final model.

**Table 4:** Distribution of basic variables of study subjects based on participation in Ageless Gym.

Variable	Participation in integrated screening Ershui township sessions (*n* = 1896)		Participation in Ageless Gym			
			Participated (*n* = 445) (23.5%)		Not participated (*n* = 1451) (76.5%)	
	*N*	%	*N*	%	*N*	%
Demographic variables						
Gender						
Female	1096	57.8	275	25.1	821	74.9
Male	800	42.2	170	21.3	630	78.8
Age						
60–69	1514	79.9	359	23.7	1155	76.3
70–79	320	16.9	76	23.8	244	76.3
≥ 80	62	3.3	10	16.1	52	83.9
Education level						
Elementary school and below	1051	55.4	251	23.9	800	76.1
Middle and high school	690	36.4	148	21.4	542	78.6
College and above	155	8.2	46	29.7	109	70.3
Lifestyle habits						
Smoking						
Never	1436	75.7	363	25.3	1073	74.7
Quit	224	11.8	52	23.2	172	76.8
Current smoker	236	12.4	30	12.7	206	87.3
Alcohol consumption						
Never	1472	77.6	348	23.6	1124	76.4
Alcohol consumption	67	3.5	13	19.4	54	80.6
Current drinker	169	8.9	36	21.3	133	78.7
Occasional drinker	188	9.9	48	25.5	140	74.5
Betel nut chewing						
Never	1686	88.9	404	24.0	1282	76.0
Quit	168	8.9	34	20.2	134	79.8
Current chewer	42	2.2	7	16.7	35	83.3
Exercise						
None	952	50.2	197	20.7	755	79.3
< 150 min/week	470	24.8	120	25.5	350	74.5
≥ 150 min/week	474	25.0	128	27.0	346	73.0
Health status						
Diabetes						
No	1614	85.1	344	21.3	1270	78.7
Yes	282	14.9	101	35.8	181	64.2
Hypertension						
No	1203	63.4	257	21.4	946	78.6
Yes	693	36.6	188	27.1	505	72.9
Heart disease						
No	1724	90.9	399	23.1	1325	76.9
Yes	172	9.1	46	26.7	126	73.3
Hepatitis						
No	1830	96.5	427	23.3	1403	76.7
Yes	66	3.5	18	27.3	48	72.7
Kidney disease						
No	1858	98.0	434	23.4	1424	76.6
Yes	38	2.0	11	28.9	27	71.1
Stroke						
No	1861	98.2	440	23.6	1421	76.4
Yes	35	1.8	5	14.3	30	85.7
Osteoporosis						
No	1793	94.6	403	22.5	1390	77.5
Yes	103	5.4	42	40.8	61	59.2
Asthma						
No	1863	98.3	439	23.6	1424	76.4
Yes	33	1.7	6	18.2	27	81.8
Hyperlipidemia						
No	1767	93.2	407	23.0	1360	77.0
Yes	129	6.8	38	29.5	91	70.5
Chronic diseases						
None	923	48.7	180	19.5	743	80.5
1 condition	305	16.1	81	26.6	224	73.4
≥ 2 conditions	668	35.2	184	27.5	484	72.5
Dietary habits						
Vegetable intake^a^						
Unhealthy	194	10.2	34	17.5	160	82.5
Healthy	1702	89.8	411	24.1	1291	75.9
Dairy products^b^						
Unhealthy	1421	74.9	341	24.0	1080	76.0
Healthy	474	25.0	104	21.9	371	78.3
Egg products^b^						
Unhealthy	1758	92.7	415	23.6	1343	76.4
Healthy	138	7.3	30	21.7	108	78.3
Fruits						
Unhealthy	1280	67.5	297	23.2	983	76.8
Healthy	616	32.5	148	24.0	468	76.0
Screening tests						
BMI						
< 24	680	35.9	138	20.3	542	79.7
≥ 24	1216	64.1	307	25.2	909	74.8
Blood pressure^c^						
Normal	395	20.8	70	17.7	325	82.3
Abnormal	1501	79.2	375	25.0	1126	75.0
Fasting blood sugar^d^						
Normal	1027	54.3	196	19.1	831	80.9
Abnormal	863	45.7	246	28.5	617	71.5
Missing	6		3	50.0	3	50.0
Waist circumference^e^						
Normal	743	39.2	143	19.2	600	80.8
Abnormal	1153	60.8	302	26.2	851	73.8
Triglycerides (TG)^f^						
Normal	1233	65.2	277	22.5	956	77.5
Abnormal	657	34.8	165	25.1	492	74.9
Missing	6		3	50.0	3	50.0
High-density lipoprotein^g^						
Normal	1070	56.6	226	21.1	844	78.9
Abnormal	820	43.4	216	26.3	604	73.7
Missing	6		3	50.0	3	50.0
Metabolic syndrome						
No	928	48.9	183	19.7	745	80.3
Yes	968	51.1	262	27.1	706	72.9
Others						
Distance from health center^h^						
Adjacent village	497	26.2	145	29.2	352	70.8
Nearby village	668	35.2	158	23.7	510	76.3
Distant village	731	38.6	142	19.4	589	80.6
Health checkup within 1 year^i^						
No	1758	92.7	399	22.7	1359	77.3
Yes	138	7.3	46	33.3	92	66.7
Participation in comprehensive screening^j^						
Once	904	47.7	169	18.7	735	81.3
Twice	508	26.8	117	23.0	391	77.0
Thrice	285	15.0	79	27.7	206	72.3
Four or more	199	10.5	80	40.2	119	59.8

^a^Vegetable intake refers to the daily intake of vegetables.

^b^Dairy products refers to the frequency of consuming milk, soy milk, or yogurt per week.

^c^Abnormal blood pressure is defined as systolic blood pressure ≥ 130 mmHg or diastolic blood pressure ≥ 85 mmHg, or currently taking antihypertensive medication.

^d^Elevated blood sugar is defined as fasting blood sugar ≥ 100 mg/dL, or currently taking diabetes medication.

^e^Abnormal waist circumference, indicator of abdominal obesity, defined as waist circumference ≥ 90 cm for men and ≥ 80 cm for women.

^f^High triglycerides are defined as blood triglyceride concentration ≥ 150 mg/dL, or currently taking medication for high triglycerides.

^g^Low high-density lipoprotein (HDL) cholesterol is defined as HDL cholesterol concentration < 40 mg/dL for men and < 50 mg/dL for women.

^h^Distance from health center: adjacent villages: approximately 1000 m, nearby villages: between 1000 and 3000 m, and distant villages: more than 3000 m.

^i^Health checkup in the past year refers to having undergone a health checkup within 1 year prior to participating in the integrated screening.

^j^Frequency of participation in comprehensive screening counts the number of times an individual participated in comprehensive screenings prior to joining the gym. For nongym participants, the count is up to December 31, 2021.

**Table 5:** Analysis of variables for initial and follow-up assessments.

Variable		Participation in Ageless Gym				
		Initial assessment (willing) (*n* = 324) (72.8%)		Follow-up assessment (consistent) (*n* = 121) (27.2%)		*p* value
		*N*	%	*N*	%	
Demographic variables						
Gender						
Female	275	196	71.3	79	28.7	0.3543
Male	170	128	75.3	42	24.7	
Age						
60–69	359	265	73.8	94	26.2	0.3293
≥ 70	86	59	68.6	27	31.4	
Education level						
Elementary school and below	251	187	74.5	64	25.5	0.3612
Middle school and above	194	137	70.6	57	29.4	
Lifestyle habits						
Smoking						
Never	363	260	71.6	103	28.4	0.2377
Former	82	64	78.0	18	22.0	
Alcohol						
Never	348	253	72.7	95	27.3	0.9229
Former	97	71	73.2	26	26.8	
Betel nut						
Never	404	294	72.8	110	27.2	0.9564
Former	41	30	73.2	11	26.8	
Exercise						
No	197	157	79.7	40	20.3	0.0018
< 150 min/week	120	88	73.3	32	26.7	
≥ 150 min/week	128	79	61.7	49	38.3	
Health status						
Diabetes						
No	344	263	76.5	81	23.5	0.0014
Yes	101	61	60.4	40	39.6	
Hypertension						
No	257	194	75.5	63	24.5	0.1378
Yes	188	130	69.1	58	30.9	
Heart disease						
No	399	291	72.9	108	27.1	0.8633
Yes	46	33	71.7	13	28.3	
Hepatitis						
No	427	309	72.4	118	27.6	0.3056
Yes	18	15	83.3	3	16.7	
Stroke						
No	440	320	72.7	120	27.3	0.7163
Yes	5	4	80.0	1	20.0	
Osteoporosis						
No	403	293	72.7	110	27.3	0.8783
Yes	42	31	73.8	11	26.2	
Asthma						
No	439	319	72.7	120	27.3	0.5597
Yes	6	5	83.3	1	16.7	
Hyperlipidemia						
No	407	300	73.7	107	26.3	0.1621
Yes	38	24	63.2	14	36.8	
Chronic diseases						
None	180	144	80.0	36	20.0	0.0193
1 condition	81	55	67.9	26	32.1	
≥ 2 conditions	184	125	67.9	59	32.1	
Dietary habits						
Vegetable intake						
Unhealthy	34	30	88.2	4	11.8	0.0354
Healthy	411	294	71.5	117	28.5	
Dairy products						
Unhealthy	341	257	75.4	84	0.7	0.0281
Healthy	104	67	64.4	37	0.3	
Egg products						
Unhealthy	415	301	72.5	114	27.5	0.6229
Healthy	30	23	76.7	7	23.3	
Fruits						
Unhealthy	297	222	74.7	75	25.3	0.1929
Healthy	148	102	68.9	46	31.1	
BMI						
< 24	138	101	73.2	37	26.8	0.904
≥ 24	307	223	72.6	84	27.4	

**Variable**		**Mean**	**Standard deviation**	**Mean**	**Standard deviation**	***p* value**

Fitness variables^a^						
Body condition						
Triceps fat thickness		7.24	3.3	7.29	3.1	0.8833
BMI		5.02	3.2	4.41	2.9	0.0678
Upper limb strength						
Grip strength		6.00	2.8	6.18	2.8	0.5377
Biceps curl		6.52	2.7	7.19	2.7	0.0226
Lower limb strength						
Sit and stand		5.66	2.7	5.83	2.9	0.5651
Upper body flexibility						
Back scratch		5.00	2.9	5.88	2.8	0.0043
Lower body flexibility						
Forward bend		5.69	2.9	6.66	2.9	0.0017
Cardiopulmonary						
2-min step		5.97	2.9	6.42	3.0	0.1478
Overall						
Up-and-go		5.30	3.0	5.32	3.1	0.9497
One-leg stand		4.13	3.2	4.18	3.1	0.8761
Total score		56.74	15.7	59.51	15.7	0.1038

^a^Fitness test scores: The results are quantified as 2 to 10 points, ranging from poor, fair, average, and good to excellent.

## Data Availability

The data that support the findings of this study are available from the Changhua County Community Integrated Screening Database, Ershui Township Health Clinic medical records, and Ageless Gym records. The data are not publicly available due to privacy or ethical restrictions, but they are available from the corresponding author upon reasonable request and with the permission of the Changhua County Health Bureau.
